# Metal Oxide Nanoparticle-Decorated Few Layer Graphene Nanoflake Chemoresistors for the Detection of Aromatic Volatile Organic Compounds

**DOI:** 10.3390/s20123413

**Published:** 2020-06-17

**Authors:** Syrine Behi, Nadra Bohli, Juan Casanova-Cháfer, Eduard Llobet, Adnane Abdelghani

**Affiliations:** 1 Research Unit of Nanobiotechnology and Valorisation of Medicinal Phytoressources UR17ES22, National Institute of Applied Science and Technology, Carthage University, Centre Urbain Nord, 1080 Charguia CEDEX Bp 676, Tunisia; syrine.behi11@gmail.com (S.B.); nadra.bohli@insat.u-carthage.tn (N.B.); adnane.abdelghani@insat.rnu.tn (A.A.); 2 Department of Electronics Engineering, Universitat Rovira i Virgili, MINOS-EMaS, 43007 Tarragona, Spain; juan.casanova@urv.cat

**Keywords:** aromatic VOC, sensor array, graphene, metal oxide nanoparticles, tin oxide, tungsten oxide, BTX

## Abstract

Benzene, toluene, and xylene, commonly known as BTX, are hazardous aromatic organic vapors with high toxicity towards living organisms. Many techniques are being developed to provide the community with portable, cost effective, and high performance BTX sensing devices in order to effectively monitor the quality of air. In this paper, we study the effect of decorating graphene with tin oxide (SnO_2_) or tungsten oxide (WO_3_) nanoparticles on its performance as a chemoresistive material for detecting BTX vapors. Transmission electron microscopy and environmental scanning electron microscopy are used as morphological characterization techniques. SnO_2_-decorated graphene displayed high sensitivity towards benzene, toluene, and xylene with the lowest tested concentrations of 2 ppm, 1.5 ppm, and 0.2 ppm, respectively. In addition, we found that, by employing these nanomaterials, the observed response could provide a unique double signal confirmation to identify the presence of benzene vapors for monitoring occupational exposure in the textiles, painting, and adhesives industries or in fuel stations.

## 1. Introduction

Industrial and human developments have been steadily increasing the production and the emission towards the environment of toxic and hazardous gases. Among the various types of pollutants, aromatic volatile organic compound vapors like benzene, toluene, and xylenes are widely used in numerous fields, most notably by the petrochemical and textile industries. According to the World Health Organization (WHO), a long-duration exposure to such compounds, especially to benzene, leads to life-threatening diseases [1]. As those vapors are harmful to both human health and the environment, the European parliament has stated via a council directive that the limit value for exposure to benzene [2] is 1.6 ppb. In addition, the US Occupational Safety and Health Administration has set permissible exposure limits (PLE), eight-hour time weighted average (TWA) concentrations for toluene and xylene, which are 10 ppm and 100 ppm, respectively [3,4]. Even though the human olfactory system can identify the characteristic odor of aromatic volatile compounds, the olfactory threshold in humans is well above the exposure limits described above. Furthermore, the olfactory system fails at correctly identifying the type of aromatic compound that may be present in a given environment. Taken together, these arguments support the idea that it is crucial to develop efficient ways for the accurate detection and monitoring of these vapors in terms of high sensitivity, good selectivity, low power consumption, low cost, and reversibility [5].

Optical based sensors, multisensor arrays, and chemoresistive sensors are among the most studied approaches for the detection of volatile organic compounds (VOCs) in general, as well as benzene, toluene, and xylene (BTX) in particular [6,7,8]. They offer suitable alternatives to the classical, expensive, bulky, and time-consuming BT detection techniques like chromatography and mass spectrometry [9]. Despite the research efforts made, inexpensive sensors still suffer from selectivity and power issues, which have hampered the development of reliable, portable, and in-field applicable BTX detectors.

Chemoresistive devices detect and identify gases by converting a gas volume fraction into an analog electrical resistance change, resulting from the charge transfer between the surface of the sensing film and the adsorbed gas molecules [10].

Nanomaterials, such as metal oxides, carbon nanotubes along with graphene and its derivatives, are among the most studied materials for the development of chemoresistive gas sensors [11,12,13].

Graphene was discovered by Novoselov and Geim [14] and over the last two decades it has been used in many applications such as electronic devices, aerospace applications, energy storage [15,16], and more. Recently, it has received much attention as gas sensitive nanomaterial due to its unique and outstanding properties such as high charge carrier mobility, low noise, and high surface to volume ratio [17,18,19,20]. Its surface can be easily modified by grafting functional groups, thus enhancing its potential as gas sensing material. However, graphene nanomaterials have also some shortcomings in gas sensing applications such as low selectivity and rather long response and recovery times [21].

To overcome these, the decoration of graphene with metal or metal oxide nanoparticles or by grafting functional groups to its surface has been explored in order to improve the performance of graphene gas sensors [22].

Moreover, other researchers worked on the improvement of gas sensors performance such as selectivity and sensitivity with the possibility of small scale integration [23,24,25,26,27]. For instance, Shi et al. [28] developed cross-reactive arrays composed of metal oxide semiconductor and graphene sensors able to discriminate similar chemical vapors (methylene, o-xylene, and toluene). Quite recently, different authors have reported tungsten or tin oxide nanoparticle-loaded graphene (or graphene derivatives such as graphene oxide or reduced graphene oxide) for enhancing the response towards different gaseous species. Tungsten oxide-loaded graphene has been found useful at detecting alcohols [29], triethylamine [30], and aniline [31]. Tin oxide-loaded graphene has been reported for the detection of, mainly, nitrogen dioxide [32,33,34,35,36], but also of formaldehyde [37,38], acethylene [39], and acetone [40].

Chemoresistive gas sensors for detecting BTX aromatic volatile compounds (benzene, toluene, xylenes) are challenging devices due to the relatively low chemical reactivity of BTX vapors [3]. In this paper, we report the response of modified graphene sensors to BTX vapors and we study the effect of the decoration of graphene with tin and tungsten oxide nanoparticles on sensitivity and selectivity. To the best of our knowledge, the presented research has not been studied before.

## 2. Materials and Methods

### 2.1. Materials

The graphene used was purchased from Strem Chemicals Inc. (Newburyport, MA, USA), with reference no. 06-0235. This material consists of graphene nanoplatelets with an average thickness of 5–10 nanometers (3.35A between sheets) and sub-micron size (a few hundreds of nanometers). In addition, its surface area is 750 m^2^/g. Commercially available nanopowders of tungsten oxide (WO_3_) and tin oxide (SnO_2_) were purchased from Sigma Aldrich (Saint Louis, MO, USA). The carrier gas was nitrogen U (N_2_ purer than 99.995%) and was bought from Air Liquide (Tunis, Tunisia). All these products were of analytical grade and used without further purification.

### 2.2. Sensing Layer Preparation

**Figure 1:**
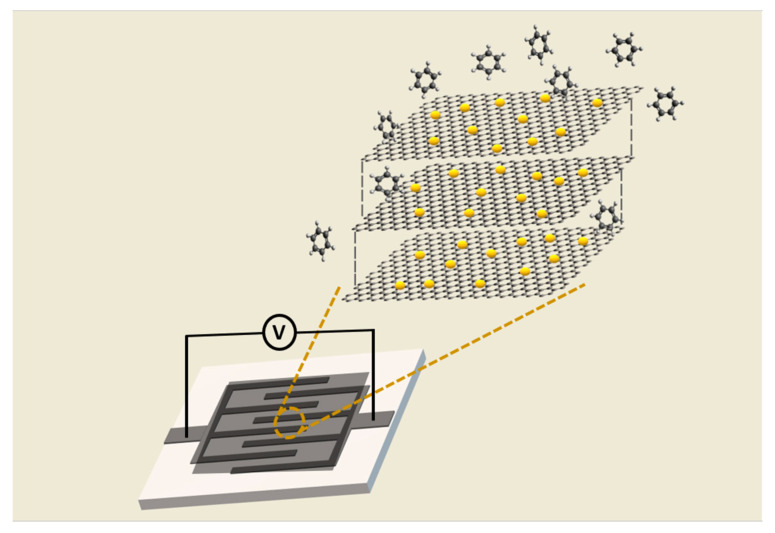
Sensor configuration: A film consisting of a random organization of few layer graphene flakes, decorated with metal oxide nanoparticle coats, and interdigitated electrodes screen-printed onto an alumina substrate. The resistance of the film is measured between the two contact pads printed on the substrate. The substrate includes a printed platinum heating meander on its backside that can be used to raise the operating temperature of the sensor above the ambient temperature.

The graphene to be used as a sensing material was prepared by chemical impregnation. In the first step, a graphene suspension was produced with ethanol (10 mg of graphene were added to 10 mL of ethanol). Then the suspension was ultrasonicated (Bandelin electronic GmbH, Berlin, Germany) for one hour in order to have a homogenous dispersion of the graphene platelets in the ethanol solution, avoiding the agglomeration of graphene. In the second step, other independent dispersions in ethanol with metal oxide nanoparticles, tin oxide (SnO_2_) and tungsten (WO_3_), were prepared (0.5 mg of SnO_2_ or WO_3_ nanoparticles were dispersed in 10 mL of ethanol). Subsequently, the suspension with the de-agglomerated graphene was placed on a hot plate while agitated vigorously at 50° and then a metal oxide NP suspension was added drop-by-drop. The stirring at 50° was kept up to half an hour after the last drop of the NP suspension was incorporated. This was done to favor the homogeneous attachment of the metal oxide nanoparticles onto the graphene surface. The loading with metal oxide NPs of graphene was adjusted to 5% in weight. In the final step, the dissolution of graphene decorated with metal oxide nanoparticles was deposited on the interdigitated gold electrodes (alumina substrate) by the airbrushing technique at a temperature between 80 °C and 90 °C degrees (see Figure 1). The room-temperature resistance of the deposited graphene, SnO_2_/graphene, and WO_3_/graphene layers was typically about 1 kΩ, 11 kΩ, and 6 kΩ, respectively. For ensuring that a high reproducibility of the deposition method is achieved, the deposition times are controlled and the device resistance is monitored during deposition to ensure that the same typical resistances mentioned above are achieved. The volume of graphene nanoflakes used on the electrodes was optimized on the basis of our previous experience in the use of carbon nanomaterials [41]. A too thick graphene coating over the electrodes results in low baseline resistance and low responsiveness. In addition, the desorption of gas molecules from a thick layer during the recovery phases becomes more difficult. Conversely, the use of a too low quantity of graphene could result in non-homogeneous coatings, dramatically reducing responsiveness, and increasing noise levels. The loading level of metal oxide nanoparticles to graphene nanoflakes was optimized in part. Our previous experience with carbon nanotube sensors indicates that the optimal levels of metal or metal oxide NP loading is in the units of % in weight [42]. Here we used a loading level of 5% in weight to ensure that graphene nanoflakes could be decorated with tin or tungsten oxide NPs avoiding the occurrence of agglomerations of the metal oxide on the graphene surface.

### 2.3. Characterization Techniques

The tested graphene sensors were characterized by transmission electron microscopy (TEM) and an Environmental Scanning Electron Microscope (ESEM). The TEM morphology investigation was undertaken with a JEOL JEM 2100F (Tokyo, Japan) TEM, at 200 kV operating voltage. The Environmental Scanning Electron Microscope was realized by Carl Zeiss AG—ULTRA 55 (Oberkochen, Germany) ESEM.

### 2.4. Vapor Sensing Experimental Setup

The graphene, SnO_2_/graphene, and WO_3_/graphene sensors were tested for the detection of BTX VOCs. The sensor chamber (35 cm^3^ in volume) can house up to six sensors at a time and measurements were performed on two replicate devices for the three different sensitive nanomaterials studied, thus ensuring that the differences in response recorded were consistent. The vapors were produced by a dilution bench consisting of a chemical solvent vaporization cell and two flowmeters (with a flow rate of 200 sccm). Sensor resistance was measured via an Agilent HP 34972A Multimeter (Santa Clara, CA, USA) at a fixed operating frequency of 50 Hz. Due to the system configuration, the lowest achievable vapor concentrations were 2, 0.4, and 0.2 ppm for benzene, toluene, and xylene, respectively.

The target vapors were injected in the sensor chamber using nitrogen as carrier gas. The response of the sensors was calculated according to the normalized resistance variation, presented in Equation (1).

∆R/R_0_ (%) = [(R_g_ − R_0_)/R_0_] × 100, (1)

where R_0_ and R_g_ are the resistance under the carrier gas and the aromatic VOC, respectively.

## 3. Results

### 3.1. Material Characterizations

#### 3.1.1. TEM Characterization

**Figure 2:**
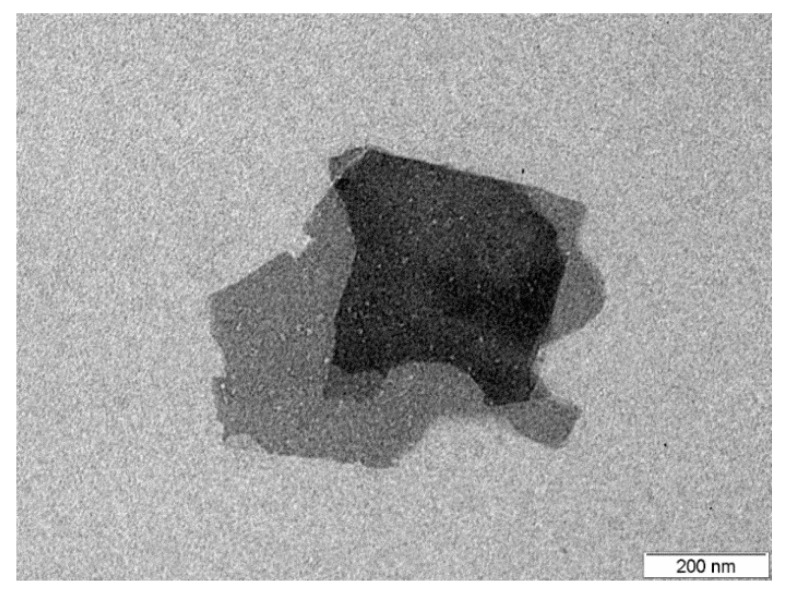
TEM image showing an example of the graphene flakes. Flakes consist of few graphene layers.

A transmission electron microscopy (TEM) analysis, performed in order to characterize the pristine graphene layers, is displayed in Figure 2.

Figure 2 shows a typical example of the 2-D graphene nanoplatelets used. They consist of few-layer graphene sheets (four layers for the sample in Figure 2) with a diameter of about a few hundreds of nanometers. This platelet shape offers a very high surface area (more than 100 m^2^/g) for its decoration with metal oxide nanoparticles and interaction with the chemical environment. Additionally, graphene has a small content of oxygen functional groups (less than 8% wt.) derived from their synthesis. Nevertheless, these oxygen groups can easily interact with metal oxide nanoparticles, enhancing the decoration of graphene.

#### 3.1.2. ESEM Characterization

**Figure 3:**
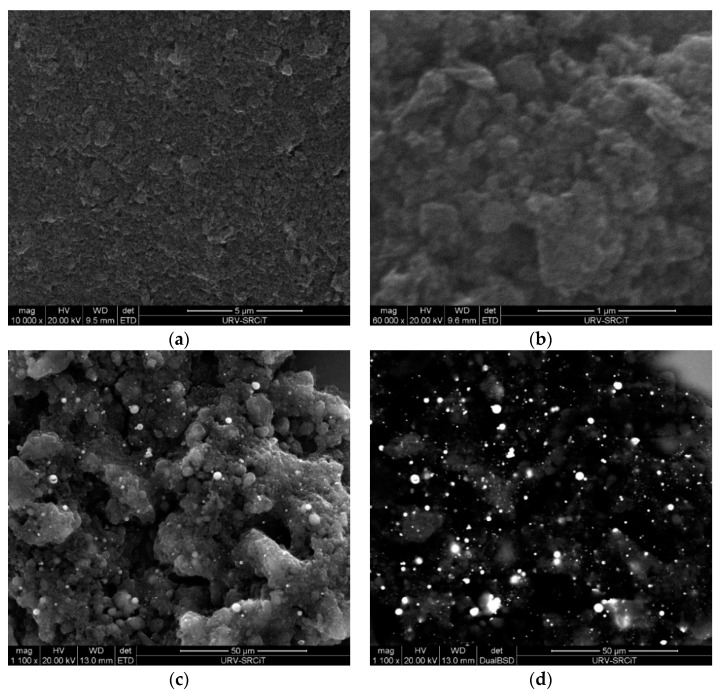

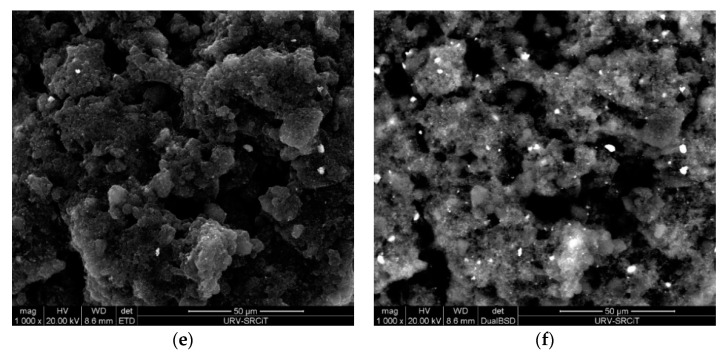
Environmental Scanning Electron Microscope (ESEM) images showing the sensor surface composed by (**a**) bare graphene (5 µm scale), (**b**) bare graphene (1 µm scale), (**c**) graphene decorated with tungsten oxide (WO_3_), (**d**) graphene decorated with WO_3_ recorded with back-scattered electron (BSE) detector, showing the graphene (black background) decorated with WO_3_ (bright spots). (**e**) Graphene decorated with tin oxide (SnO_2_), (**f**) graphene decorated with SnO_2_ recorded with back-scattered electron (BSE) detector, showing the graphene (black background) decorated with SnO_2_.

Additionally, ESEM images were obtained for pristine and graphene decorated with WO_3_ and SnO_2_. Figure 3a,b shows the ESEM images of graphene once it was deposited onto the substrate to be employed as a gas sensor at two different scales. In addition, in Figure 3c,e, the graphene decorated with WO_3_ and SnO_2_ are observed. In Figure 3d,f, a back-scattered electron detector (BSE) was used showing the graphene (black background) decorated with the metal oxide nanoparticles, which correspond to the bright spots. These images reveal a quite homogeneous distribution of WO_3_ and SnO_2_ metal oxide nanoparticles on the graphene surface.

### 3.2. Vapor Detection

The sensing performances of the pristine graphene, SnO_2_/graphene, and WO_3_/graphene were examined for aromatic vapors: benzene (C_6_H_6_, dipolar moment µ = 0), toluene (C_6_H_7_, dipolar moment µ = 0.43), and o-xylene (C_8_H_10_, dipolar moment µ = 0.62). The vapor concentration was controlled by changing the mixing ratio of nitrogen and nitrogen balanced analyte vapor using mass flow controllers.

#### 3.2.1. Graphene Sensing Response to Aromatic VOCs

**Figure 4:**
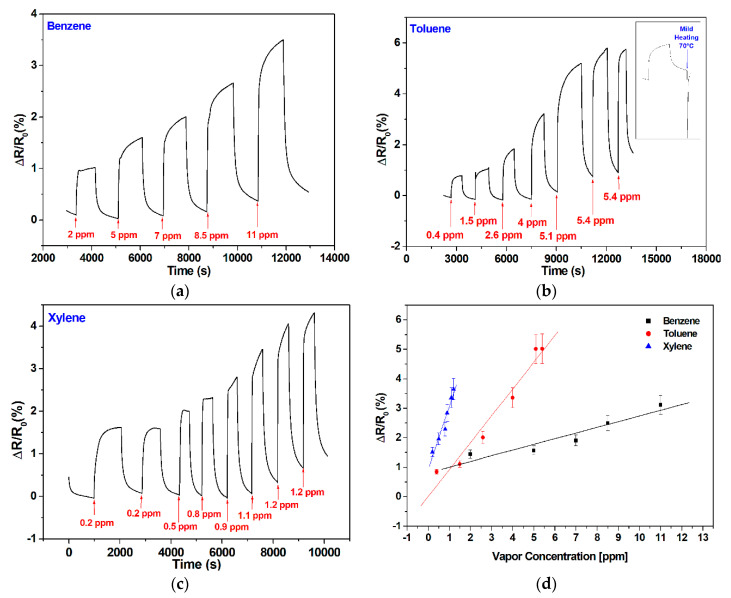
Graphene sensor responses for different concentrations of injected vapors of (**a**) benzene; (**b**) toluene; (**c**) xylene; and (**d**) associated calibration curves. The inset in panel b shows how applying mild heating helps regenerating sensor surface and fully recovering its baseline.

The vapors sensing performances at room temperature of the pristine graphene-based sensor towards the aromatic vapors benzene, toluene, and xylene are presented in Figure 4.

The observed response of the graphene-based sensor shows an increase of the sensor resistance with the increase in the injected vapor concentration either for benzene, toluene, or xylene. A good repeatability was observed (Figure 4b,c) at room temperature. The mechanisms behind the responses registered are probably based on electrostatic interactions. Thereby, even though π–π interactions cannot be ruled out, the room-temperature desorption of gas molecules indicates that the formation of H-bonds is probably the most predominant interaction.

A baseline drift was observed for the higher concentration tested. Due to the highly stable and reversible responses recorded for the lowest concentrations tested, this effect can be attributed to VOC molecules remaining adsorbed at the graphene surface at the end of a cleaning phase, preventing a complete baseline recovery. In fact, a short heating pulse of a few seconds at 70 °C is enough for completely desorbing all vapor molecules and fully regaining the baseline (see the inset in Figure 4b). This heating was obtained by applying an external voltage (one volt) to a heating element printed on the backside of the sensor substrate and helps desorbing vapor molecules from the graphene surface. The same behavior has been observed in a previous research in which carbon nanotubes were used [43]. This strategy of heating was applied only once to show its effect, but all the measurements shown in Figure 4 were performed at room temperature and no heating was used to help regain the baseline. The lowest concentration tested were 2 ppm, 0.4 ppm, and 0.2 ppm for benzene, toluene, and xylene, respectively, which, combined to the very low noise levels observed in these responses (0.2% at room temperature), indicates that the lower detection limit is in a range of about 200 ppb for benzene and toluene and about 50 ppb for xylene. This estimation of the limit of detection (LOD) considers that a meaningful response signal should be at least three times higher than the noise level experienced. The calibration curves presented in Figure 4d reveal a better sensitivity (i.e., slope of the calibrations curves) for xylene compared to benzene and toluene. The error bars in the calibration curves shown were calculated as the mean +/− the standard deviation over replicate measurements performed with two replicate bare graphene sensors.

It is noteworthy to stress that the best graphene sensor performance, either from its sensitivity or its baseline stability, was observed when operated at room temperature. Additional measurements performed at operating temperatures above the ambient indicate that the response decreases when graphene is heated. This decrease in responsiveness is probably due to the fact that heating promotes desorption of the weakly physisorbed VOC molecules from the graphene surface. Such results are presented in the Supplementary Materials file, Figure S1 and Table S1.

#### 3.2.2. Sensing Response to Aromatic VOCs of Metal Oxide Nanoparticle-Decorated Graphene

It is well known that semiconductor metal oxide materials such as tin or tungsten oxides can be used as sensitive materials for the detection of vapors and gases [44,45,46]. Here we studied the effect of decorating graphene with nanoparticles of either tungsten or tin oxide for tuning gas sensing properties. First, an optimization of the sensor operating temperature was conducted by characterizing sensor response at different temperatures ranging from room temperature to 300 °C. It was observed that the response of WO_3_/graphene and SnO_2_/graphene sensors towards the VOCs tested increased when the working temperature was raised up to 250 °C and then decreased again at 300 °C. The substantial increase in the optimal operating temperature of hybrid sensors in comparison to the one of bare graphene is because metal oxide nanoparticles need such high temperatures for activating the chemisorption and reaction of VOCs. However, a too high operating temperature hinders the adsorption of gas molecules and results in decreased responsiveness [47]. The results on the performance of metal oxide-decorated graphene sensors as a function of the operating temperature can be found in the Supplementary Materials file, Figures S2–S6 and Tables S2 and S3. The following sub-sections show the results obtained when sensors were operated at their optimal working temperature of 250 °C.

##### Response of Tungsten Oxide Nanoparticle-Decorated Graphene

**Figure 5:**
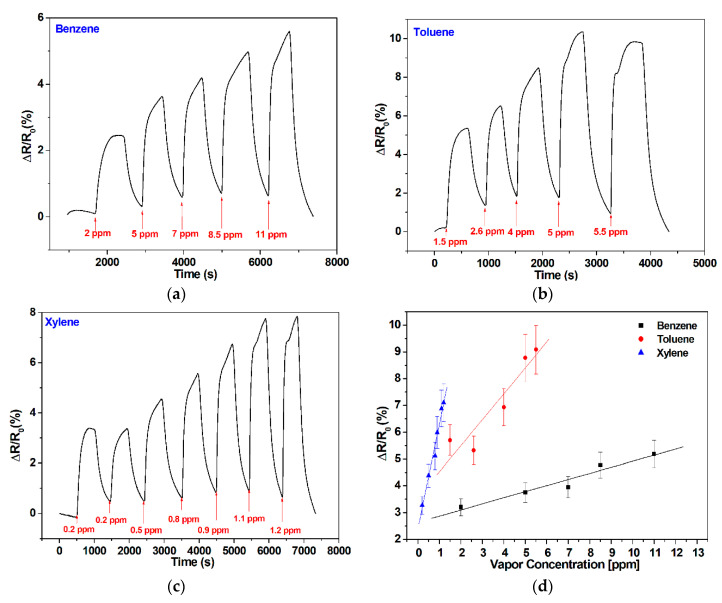
WO_3_/graphene sensor responses for different concentrations of injected vapors of (**a**) benzene; (**b**) toluene; (**c**) xylene; and (**d**) associated calibration curves. For the responses shown in panels a, b, and c, a linear baseline correction has been implemented (see Supplementary Materials for more details).

The responses of the WO_3_/graphene-based sensor operated at 250 °C towards the vapors of benzene, toluene, and xylene are presented in Figure 5.

The observed response of the WO_3_/graphene-based sensor shows an increase in the sensor resistance with the increase of the injected vapor concentration, for any of the aromatic VOCs tested. A good repeatability was observed (Figure 5c) at 250 degrees. The mechanisms behind the responses correspond to an overall p-type semiconductor behavior of the gas sensitive film. The chemisorption of aromatic VOCs onto the tungsten oxide nanoparticles transfers electrons from the adsorbed molecule towards the conduction band of tungsten oxide. These electrons are eventually injected to graphene, shifting the Fermi level of graphene towards the conduction band. As a result, the number of holes in graphene decreases and the overall resistance increases [48]. The calibration curves presented in Figure 5d reveal a better sensitivity for xylene compared to benzene and toluene. The error bars in the calibration curves shown were calculated as the mean +/− the standard deviation over replicate measurements performed with two replicate WO_3_/graphene sensors. Once again, the lowest concentration tested were 2 ppm, 1.5 ppm, and 0.2 ppm for benzene, toluene, and xylene, respectively, which, combined to the very low noise levels observed in these responses, indicates that the lower detection limit for the aromatic VOCs is about 100 ppb for benzene and toluene and about 20 ppb for xylene. The lower detection limit was estimated in relation to the observed response noise of 0.3% at 250 °C. The response signal at these low concentrations is higher (roughly two times higher) than the one recorded with bare graphene. This implies that the limit of detection for VOCs in tungsten oxide-decorated graphene sensors is better (i.e., lower) than in bare graphene sensors.

##### Response of Tin Oxide Nanoparticle-Decorated Graphene

**Figure 6:**
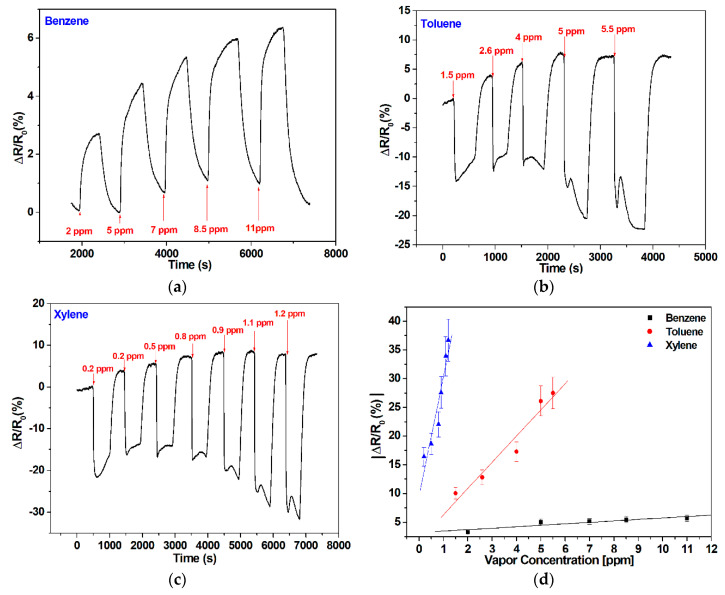
SnO_2_/graphene sensor responses for different concentrations of injected vapors of (**a**) benzene; (**b**) toluene; (**c**) xylene; and (**d**) associated calibration curves. A linear baseline correction was implemented for benzene exclusively (response shown in panel a), (see Supplementary Materials for more details).

The responses of the SnO_2_/graphene-based sensor operated at 250 °C towards the vapors of benzene, toluene, and xylene are presented in Figure 6.

For benzene vapors, the SnO_2_/graphene-based sensor shows the same p-type behavior already observed in bare and tungsten oxide-decorated graphene. Sensor resistance increases for increasing concentrations of benzene. However, for toluene and xylene vapors, the sensor behaves as an n-type semiconductor, because its resistance clearly decreases for increasing concentrations of these species. Such transitions from p-type to n-type materials have been reported before in graphene nanomaterials and attributed to adsorbate induced charge transfer and charge carrier scattering effects [49,50]. These response inversion effects appear at different operating temperatures for tin oxide and tungsten oxide-decorated graphene, and the dipole moment (DM) of the molecule detected plays an important role. Indeed, for SnO_2_/graphene-based sensors, while this effect is not seen for benzene (DM = 0), it is observed for toluene and o-xylene, which have increasing DM. For ethanol vapors, a molecule with even higher DM, this response inversion effect appears not only in SnO_2_/graphene but also in WO_3_/graphene operated at 300 °C (these results are summarized in the Supplementary Materials).

The calibration curves presented in Figure 6d reveal a better sensitivity for xylene compared to benzene and toluene. The error bars in the calibration curves shown were calculated as the mean +/− the standard deviation over replicate measurements performed with two replicate SnO_2_/graphene sensors. Once more, the very low noise levels observed in the responses indicate that the lower detection limit for the aromatic VOCs lies in the range of a few hundred of ppb (i.e., about 100 ppb for benzene and toluene and about 20 ppb for xylene). The lower detection limit was estimated in relation to the observed response noise of 0.3% at 250 °C. A good repeatability was observed (see Figure 6c) at 250 degrees. The mechanism behind the response signals at these low concentrations is even higher than the one recorded with WO_3_/graphene. This implies that the limit of detection for VOCs in SnO_2_/graphene sensors is better (i.e., lower) than in tungsten WO_3_/graphene and bare graphene sensors.

### 3.3. Sensitivity to Aromatic VOCs

To calculate the sensitivities of the fabricated sensors to all the tested vapors, the calibration curves were fitted using a linear regression model. The sensitivity (slopes of the calibration curves) for pristine graphene, WO_3_/graphene, and SnO_2_/graphene sensors are presented in Table 1.

The best performance is observed for the SnO_2_/graphene sensor for benzene, toluene, and xylene vapors. These differences can be related to the different dipolar moments of the aromatic VOCs tested and the different activation energy of the gas-sensitive materials. The higher sensitivity for tin oxide-decorated graphene could also be due to a better matching between the work function of graphene (near 4.6 eV) and that of tin oxide (near 4.5 eV) as compared to tungsten oxide (near 5 eV) [51,52,53]. Results on the long-term stability of sensors are shown in the Supplementary Materials. A 20% drop in sensitivity was observed after one month of operation under humid conditions. The sensitivity towards non-aromatic VOCs was tested by measuring ethanol (see Figure S6, Tables S2 and S3, Supplementary Materials). Ethanol sensitivity was found to be comparable to the one for benzene but significantly lower than the one for toluene (3-fold lower) or xylene (10-fold lower). There are very few reports in the literature about the detection of aromatic VOCs using carbon nanomaterials. Table 2 summarizes a comparison between the performance in the detection of BTX vapors reported here and previously published results. The normalized response of our metal oxide-decorated graphene sensors show higher values for xylene and toluene vapors than those reported in previously published results [3,41,42,43,54]. Mirzaei and co-workers report in their review [3] a ZnO nanowire sensor modified with small amounts of reduced graphene oxide (rGO) with a remarkably high response to benzene vapors. This differs from our approach in which graphene is modified with small amounts of metal oxides. However, the rGO-loaded metal oxide sensor reported operates at rather high temperatures and is loaded with Pd [3]. At such temperatures, Pd acts as a catalyst and high cross-sensitivity to hydrogen, carbon monoxide, alcohols, or hydrocarbons can be foreseen.

It is worth mentioning that the loading level of metal oxide nanoparticles on graphene could be optimized further in order to possibly achieve an even higher sensitivity to aromatic VOCs. This would involve checking different loading levels in a narrow range near the 5% level used here. Loading levels below 1% do not significantly alter the properties of the bare material and loading levels higher than 10% result in the agglomeration of metal oxide nanoparticles, inhomogeneous distribution of these on the graphene surface, and loss of sensitivity.

### 3.4. Response Time and Recovery Time

The response and recovery times of graphene, SnO_2_/graphene, and WO_3_/graphene sensors toward aromatic vapors are presented in Table 3 and Table 4, respectively. The response time was estimated as the time required for the sensor to respond from 10% to 90% of its maximum response value. The recovery time was estimated as the time required for the sensor signal to fall towards its baseline, from 90% to 10% of its response value during a cleaning step. The results show faster response times for the SnO_2_/graphene sensor towards toluene and xylene, roughly half a minute. These response times compare favorably to the reported results for graphene sensors [3,48]. For benzene, the best response time is displayed by the graphene sensor. The recovery times of the developed sensors range between 2.5 min and nearly 7 min. A rather slow return to the baseline is achieved compared to metal oxide chemoresistors [3]. This is possibly due to the strong interaction between VOC molecules and the surface of metal oxide-loaded graphene sensors and the lower operating temperatures used.

## 4. Discussion

It is well known that the exposure to high concentrations of BTX vapors, commonly used as organic solvents in the industrial production of paints, lacquers, adhesives, and detergents may cause serious carcinogenic effects. The monitoring of the occupational exposure to these compounds is thus of great importance for the preservation of workers’ health. Currently, the eight-hour TVA level for benzene as indicated by OSHA is 1 ppm (the TVA levels for toluene and xylene are higher) [55]. The metal oxide-loaded graphene sensors reported here are sensitive enough and show promising selectivity for detecting BTX vapors at the relevant concentrations for monitoring occupational exposure. The results reported here can be used to further the progress towards the development of a sensor array for the selective detection of BTX.

In a previous research [54], we showed that a sensor employing multiwall carbon nanotubes (MWCNTs) decorated with gold nanoparticles and functionalized with a self-assembled monolayer of long-chain thiols (MHDA) was selective to non-aromatic vapors. This material was insensitive to aromatic VOCs, which can be used as a negative test for confirming/ruling out the presence of aromatic vapors in a complex environment. The three materials studied here (i.e., graphene and the two metal oxide-decorated graphene) display different, overlapping sensitivities for aromatic VOCs. However, the fact that tin oxide-loaded graphene shows a characteristic response inversion for toluene and xylene vapors (and not for benzene) can be used as a confirmation test for the presence of benzene in the environment analyzed. To illustrate this approach, a lack of response from the MHDA-Au-MWCNT sensor combined with p-type responses from the other three sensors would be indicative of the presence of benzene. In contrast, a lack of response from the MHDA-Au-MWCNT sensor combined with a p-type response from the graphene and WO_3_/graphene sensors together with an n-type response from the SnO_2_/graphene sensor would be indicative of the presence of toluene or xylene. Finally, a response from the MHDA-Au-MWCNT sensor would indicate the presence of a non-aromatic VOC such as methanol, ethanol, or acetone.

## 5. Conclusions

In this paper, we studied the effect of tin oxide (SnO_2_) and tungsten oxide (WO_3_) nanoparticles decorating graphene on the performance of the resulting hybrid nanomaterials as chemiresistive sensors for detecting BTX vapors. The decoration of few layer graphene flakes resulted in high sensitivity towards benzene, toluene, and xylene vapors. The sensors could be suitable for the inexpensive monitoring occupational exposure to BTX. In a future research, the materials developed could be used for integrating a four-element sensor array on a single-chip MEMS configuration, and thus could provide a unique double signal confirmation response associated with the presence of highly toxic benzene vapors (as described in the discussion section). The fabrication of this single-chip sensor, the fine-tuning of the loading level of graphene with metal oxide nanoparticles to maximize the response to aromatic VOCs, and the study of the effect of ambient moisture will be the subject of our next research.

## Figures and Tables

**Table 1:** Sensor sensitivity (10^−2^ × ppm^−1^) for aromatic vapors.

	Graphene	WO_3_/Graphene	SnO_2_/Graphene
Benzene	19.2	22.7	25.5
Toluene	91	96.9	456.4
Xylene	213.6	391.2	2081.8

**Table 2:** Comparison of the normalized responses (NR) expressed as response % × ppm^−1^ (where response % is defined as Rg/R_0_ (%)) for benzene, toluene, and xylene for different carbon nanomaterial gas sensors. Other parameters such as the limit of detection (LOD) expressed in ppb, operating temperature (°C), and dynamic range of concentration measured experimentally (in ppm) are given too.

	Temp	Benzene		Toluene		Xylene		Dynamic Range	Ref.
	(°C)	NR	LOD	NR	LOD	NR	LOD	(ppm)	
Graphene	RT	0.56	200	0.95	200	4	50	0.2–11	This research
Graphene/WO_3_	250	0.96	100	2	100	7	20	0.2–11	This research
Graphene/SnO_2_	250	1.16	100	4.25	100	28	20	0.2–11	This research
Pd-rGO-ZnO	400	460	100 *	N/A	N/A	N/A	N/A	1–5	[3]
MWCNT-PEO	RT	N/A	N/A	0.003	55,000 *	N/A	N/A	72–108	[3]
MWCNT-Au-Calixarene	RT	5	0.6	0.15	100 *	0.025	200 *	0.02–0.08	[41]
MWCNT-FeO	RT	0.35	1400 *	0.45	1000 *	N/A	N/A	1.52–11.25	[42]
MWCNT-Au-HDT	RT	1.5	500 *	4	250 *	N/A	N/A	0.5–13	[43]
MWCNT-Au-MHDA	RT	0	-	0	-	N/A	N/A	5–20	[54]

*: estimated values for the LOD from data reported in the paper. N/A: data not available.

**Table 3:** Response time (s).

	Graphene	WO_3_/Graphene	SnO_2_/Graphene
Benzene	87	275	238
Toluene	113	164	26
Xylene	136	178	35

**Table 4:** Recovery time (s).

	Graphene	WO_3_/Graphene	SnO_2_/Graphene
Benzene	200	412	242
Toluene	188	285	148
Xylene	222	397	278

## Supplementary Materials

The following are available online at https://www.mdpi.com/1424-8220/20/12/3413/s1, Figure S1: Graphene sensor responses for different concentrations of injected benzene vapors at (a) room temperature, (b) 50 °C, (c) 75 °C, and (d) 100 °C, Table S1: Graphene sensitivity (10^−2^x ppm^−1^) for different vapors at different temperatures, Figure S2: Decorated graphene sensors responses for 5 ppm concentration of injected benzene vapor at (a) room temperature and (b) 80°C, Figure S3: SnO_2_/graphene and WO_3_/graphene sensor responses for different concentrations of injected benzene vapors at (a) room temperature, (b) 275 °C, and (c) 300 °C, Figure S4: SnO_2_/graphene and WO_3_/graphene sensor responses for different concentrations of injected toluene vapors at (a) room temperature, (b) 275 °C, and (c) 300 °C, Figure S5: SnO_2_/graphene and WO_3_/graphene sensor responses for different concentrations of injected xylene vapors at (a) 200 °C, (b) 275 °C, and (c) 300 °C, Figure S6: SnO_2_/graphene and WO_3_/graphene sensor responses for different concentrations of injected ethanol vapors at (a) room temperature, (b) 200 °C, (c) 250 °C, (d) 275 °C, and (e) 300 °C, Table S2: WO_3_/graphene sensitivity (10^−2^x ppm^−1^) for different vapors at different temperatures, Table S3: SnO_2_/graphene sensitivity (10^−2^x ppm^−1^) for different vapors at different temperatures. Figure S7: SnO_2_/graphene sensor responses for different concentrations of injected vapors of benzene (a) without and (b) with linear baseline correction. Figure S8: Long-term stability of the response towards benzene vapors.

## References

[1] WHO, Air Pollution and Child Health: Prescribing Clean Air. http://www.who.int/ceh/publications/air-pollution-child-health/en/.

[2] Clément P., Llobet E., Woodhead Publishing Series in Electronic and Optical Materials (2020). Carbon Nanomaterials Functionalized with Macrocyclic Compounds for Sensing Vapors of Aromatic VOCs. Semiconductor Gas Sensors.

[3] Mirzaei A., Kim J.-H., Kim H.W., Kim S.S. (2018). Resistive-Based Gas Sensors for Detection of Benzene, Toluene and Xylene (BTX) Gases: A Review. J. Mater. Chem. C.

[4] Table AC1—Permissible Exposure Limits for Chemical Contaminants. https://www.dir.ca.gov/title8/5155table_ac1.html.

[5] Wu J., Feng S., Li Z., Tao K., Chu J., Miao J., Norford L.K. (2018). Boosted Sensitivity of Graphene Gas Sensor via Nanoporous Thin Film Structures. Sens. Actuator B-Chem..

[6] Bogue R. (2015). Detecting gases with light: A review of optical gas sensor technologies. Sens. Rev..

[7] Kadir R., Yimit A., Ablat H., Mahmut M., Itoh K. (2009). Optical Waveguide BTX Gas Sensor Based on Polyacrylate Resin Thin Film. Environ. Sci. Technol..

[8] Nizamidin P., Yimit A., Nurulla I., Itoh K. (2012). Optical Waveguide BTX Gas Sensor Based on Yttrium-Doped Lithium Iron Phosphate Thin Film. Int. Sch. Res. Not..

[9] Zhang S., Zhao T., Xu X., Wang H., Miao C. (2010). Determination of BTEX Compounds in Solid–Liquid Mixing Paint Using the Combination of Solid Phase Extraction, Thermal De-sorption and GC-FID. Chromatographia.

[10] Yuan W., Shi G. (2013). Graphene-Based Gas Sensors. J. Mater. Chem. A.

[11] Xu K., Fu C., Gao Z., Wei F., Ying Y., Xu C., Fu G. (2018). Nanomaterial-Based Gas Sensors: A Review. Instrum. Sci. Technol..

[12] Han T., Nag A., Mukhopadhyay S.C., Xu Y. (2019). Carbon nanotubes and its gas-sensing applications: A review. Sens. Actuator A-Phys..

[13] Lin T., Lv X., Hu Z., Xu A., Feng C. (2019). Semiconductor Metal Oxides as Chemoresistive Sensors for Detecting Volatile Organic Compounds. Sensors.

[14] Novoselov K.S., Geim A.K., Morozov S.V., Jiang D., Katsnelson M.L., Grigorieva I.V. (2004). Electric Field Effect in Atomically Thin Carbon Films. Science.

[15] Wang B., Hu C., Dai L. (2016). Functionalized Carbon Nanotubes and Graphene-Based Materials for Energy Storage. Chem. Commun..

[16] Singh E., Meyyappan M., Nalwa H.S. (2017). Flexible Graphene-Based Wearable Gas and Chemical Sensors. ACS Appl. Mater. Interfaces.

[17] Yavari F., Koratkar N. (2012). Graphene-Based Chemical Sensors. J. Phys. Chem. Lett..

[18] Yu X., Zhang W., Zhang P., Su Z. (2017). Fabrication technologies and sensing applications of graphene-based composite films: Advances and challenges. Biosens. Bioelectron..

[19] Wrobel P.S., Wlodarski M.D., Jedrzejewska A., Placek K.M., Szukiewicz R., Kotowicz S., Tokarska K., Quang H.T., Mendes R.G., Liu Z. (2019). A comparative study on simple and practical chemical gas sensors from chemically modified graphene films. Mater. Res. Express.

[20] Wang T., Huang D., Yang Z., Xu S., He G., Li X., Hu N., Yin G., He D., Zhang L. (2016). A Review on Graphene-Based Gas/Vapor Sensors with Unique Properties and Potential Applications. Nano-Micro Lett..

[21] Tian W., Liu X., Yu W. (2018). Research Progress of Gas Sensor Based on Graphene and Its Derivatives: A Review. Appl. Sci..

[22] Gutés A., Hsia B., Sussman A., Mickelson W., Zettl A., Carraro C., Maboudian R. (2012). Graphene Decoration with Metal Nanoparticles: Towards Easy Integration for Sensing Applications. Nanoscale.

[23] Kamal Z.-E.-H., Salahuddin M.A., Benhaddou D., Al-Fuqaha A. (2015). Introduction to Wireless Sensor Networks. Wireless Sensor Networks: Architectures and Protocols.

[24] Pinnaduwage L.A., Gehl A.C., Allman S.L., Johansson A., Boisen A. (2007). Miniature Sensor Suitable for Electronic Nose Applications. Rev. Sci. Instrum..

[25] Park S.Y., Kim Y., Kim T., Eom T.H., Kim S.Y., Jang H.W. (2019). Chemoresistive materials for electronic nose: Progress, perspectives, and challenges. InfoMat.

[26] Zhang Y., Zhao J., Du T., Zhu Z., Zhang J., Liu Q. (2017). A gas sensor array for the simultaneous detection of multiple VOCs. Sci. Rep..

[27] Potyrailo R.A., Surman C., Nagraj N., Burns A. (2011). Materials and Transducers Toward Selective Wireless Gas Sensing. Chem. Rev..

[28] Shi C., Ye H., Wang H., Ioannou D.E., Li Q. (2018). Precise gas discrimination with cross-reactive graphene and metal oxide sensor arrays. Appl. Phys. Lett..

[29] Qin J., Cao M., Li N., Hu C. (2011). Graphene-wrapped WO_3_ nanoparticles with improved performances in electrical conductivity and gas sensing properties. J. Mater. Chem..

[30] Gui Y., Zhao J., Wang W., Tian J., Zhao M. (2015). Synthesis of hemispherical WO_3_/graphene nanocomposite by a microwave-assisted hydrothermal method and the gas-sensing properties to trimethylamine. Mater. Lett..

[31] Gui Y., Liu Z., Fang S., Tian J., Gong F. (2016). Synthesis of flower-like WO_3_/graphene nanocomposite by microwave-assisted hydrothermal method and the enhanced gas-sensing properties to aniline. J. Mater. Sci-Mater. Electron..

[32] Wu J., Wu Z., Ding H., Wei Y., Huang W., Yang X., Li Z., Qiu L., Wang X. (2020). Three-Dimensional Graphene Hydrogel Decorated with SnO_2_ for High-Performance NO_2_ Sensing with Enhanced Immunity to Humidity. ACS Appl. Mater. Interfaces.

[33] Wang Z., Jia Z., Li Q., Zhang X., Sun W., Sun J., Liu B., Ha B. (2019). The enhanced NO_2_ sensing properties of SnO_2_ nanoparticles/reduced graphene oxide composite. J. Colloid. Interface. Sci..

[34] Wang Z., Zhang T., Han T., Fei T., Liu S., Lu G. (2018). Oxygen vacancy engineering for enhanced sensing performances: A case of SnO_2_ nanoparticles-reduced graphene oxide hybrids for ultrasensitive ppb-level room-temperature NO_2_ sensing. Sens. Actuator B-Chem..

[35] Wang Z., Zhao C., Han T., Zhang Y., Liu S., Fei T., Lu G., Zhang T. (2017). High-performance reduced graphene oxide-based room-temperature NO_2_ sensors: A combined surface modification of SnO_2_ nanoparticles and nitrogen doping approach. Sens. Actuator B-Chem..

[36] Tammanoon N., Wisitsoraat A., Sriprachuabwong C., Phokharatkul D., Tuantranont A., Phanichphant S., Liewhiran C. (2015). Ultrasensitive NO_2_ Sensor Based on Ohmic Metal-Semiconductor Interfaces of Electrolytically Exfoliated Graphene/Flame-Spray-Made SnO_2_ Nanoparticles Composite Operating at Low Temperatures. ACS Appl. Mater. Interfaces.

[37] Bo Z., Yuan M., Mao S., Chen X., Yan J., Cen K. (2018). Decoration of vertical graphene with tin dioxide nanoparticles for highly sensitive room temperature formaldehyde sensing. Sens. Actuator B-Chem..

[38] Cao Y., Li Y., Jia D., Xie J. (2014). Solid-state synthesis of SnO_2_-graphene nanocomposite for photocatalysis and formaldehyde gas sensing. RSC Adv..

[39] Jin L., Chen W., Zhang H., Xiao G., Yu C., Zhou Q. (2017). Characterization of reduced graphene oxide (rGO)-loaded SnO_2_ nanocomposite and applications in C_2_H_2_ gas detection. Appl. Sci..

[40] Zhang D., Liu A., Chang H., Xia B. (2015). Room-temperature high-performance acetone gas sensor based on hydrothermal synthesized SnO_2_-reduced graphene oxide hybrid composite. RSC Adv..

[41] Clément P., Korom S., Struzzi C., Parra E.J., Bittencourt C., Ballester P., Llobet E. (2015). Deep Cavitand Self-Assembled on Au NPs-MWCNT as Highly Sensitive Benzene Sensing Interface. Adv. Funct. Mater..

[42] Clément P., Hafaiedh I., Parra E.J., Thamri A., Guillot J., Abdelghani A., Llobet E. (2014). Iron oxide and oxygen plasma functionalized multi-walled carbon nanotubes for the discrimination of volatile organic compounds. Carbon.

[43] Bohli N., Belkilani M., Casanova-Chafer J., Llobet E., Abdelghani A. (2019). Multiwalled carbon nanotube based aromatic volatile organic compound sensor: Sensitivity enhancement through 1-hexadecanethiol functionalization. Beilstein J. Nanotechnol..

[44] Labidi A., Jacolin C., Bendahan M., Abdelghani A., Guerin J., Aguir K., Maaref M. (2005). Impedance spectroscopy on WO3 gas sensor. Sens. Actuator B-Chem..

[45] Hafaiedh I., Helali S., Cherif K., Abdelghani A., Tournier G. (2008). Characterization of Tin Dioxide Film for Chemical Vapors sensor. Mat. Sci. Eng. C..

[46] Sun D., Luo Y., Debliquy M., Zhang C. (2018). Graphene-enhanced metal oxide gas sensors at room temperature: A review. Beilstein J. Nanotechnol..

[47] Sakai G., Matsunaga N., Shimanoe K., Yamazoe N. (2001). Theory of gas-diffusion controlled sensitivity for thin film semiconductor gas sensor. Sens. Actuator B-Chem..

[48] Deokar G., Casanova-Cháfer J., Rajput N.S., Aubry C., Llobet E., Jouiad M., Costa P.M. (2020). Wafer-scale few-layer graphene growth on Cu/Ni films for gas sensing applications. Sens. Actuator B-Chem..

[49] Pearce R., Iakimov T., Andersson M., Hultman L., Lloyd Spetz A., Yakimova R. (2011). Epitaxially grown graphene based gas sensors for ultra sensitive NO_2_ detection. Sens. Actuator B-Chem..

[50] Jaaniso R., Kahro T., Kozlova J., Aarik J., Aarik L., Alles H., Floren A., Gerst A., Kasikov A., Niilisk A. (2014). Temperature induced inversion of oxygen response in CVD graphene on SiO_2_. Sens. Actuator B-Chem..

[51] Naghdia S., Sanchez-Arriagaa G., Rheeb K.Y. (2019). Tuning the work function of graphene toward application as anode and cathode. J. Alloy. Compd..

[52] Halek G., Baikie I.D., Teterycz H., Halek P., Suchorska P., Wiśniewski K. (2013). Work Function Analysis of Gas Sensitive WO_3_ Layers with Pt Doping. Sens. Actuator B-Chem..

[53] Schierbaum K.D., Weimar U., Göpel W., Kowalkowski R. (1991). Conductance, Work Function and Catalytic Activity of SnO_2_-Based Gas Sensors. Sens. Actuator B-Chem..

[54] Thamri A., Baccar H., Struzzi C., Bittencourt C., Abdelghani A., Llobet E. (2016). MHDA-functionalized multiwall carbon nanotubes for detecting non-aromatic VOCs. Sci. Rep..

[55] Occupational Safety and Health Administration, Substance Safety Data Sheet, Benzene. https://www.osha.gov/laws-regs/regulations/standardnumber/1910/1910.1028AppA.

