# Supplementary material for: Metal Oxide Nanoparticle-Decorated Few Layer Graphene Nanoflake Chemoresistors for the Detection of Aromatic Volatile Organic Compounds
Source: Sensors (Basel). 2020 Jun 17;20(12):3413. doi: 10.3390/s20123413 (PMC7349069; doi:10.3390/s20123413)
Supplement: Supplementary file 1 [file sensors-20-03413-s001.pdf]

Supplementary Material for

Metal oxide nanoparticle decorated few layer graphene nanoflake chemoresistors for detecting aromatic volatile organic compounds.

Syrine Behi [1], Nadra Bohli [1], Juan Casanova-Cháfer [2], Eduard Llobet [2,*] and Adnane Abdelghani [1]

[1] Carthage University, National Institute of Applied Science and Technology, Research Unit of Nanobiotechnology and Valorisation of Medicinal Phytoressources UR17ES22, Bp 676, Centre Urbain Nord, 1080 Charguia Cedex, Tunisia; nadra.bohli@insat.u-carthage.tn

[2] Universitat Rovira i Virgili, MINOS-EMaS, 43007 Tarragona, Spain; eduard.llobet@urv.cat

* Correspondence: eduard.llobet@urv.cat

Response of bare graphene towards benzene vapors at different operating temperatures. These results show that the optimal working temperature for bare graphene is at room temperature.

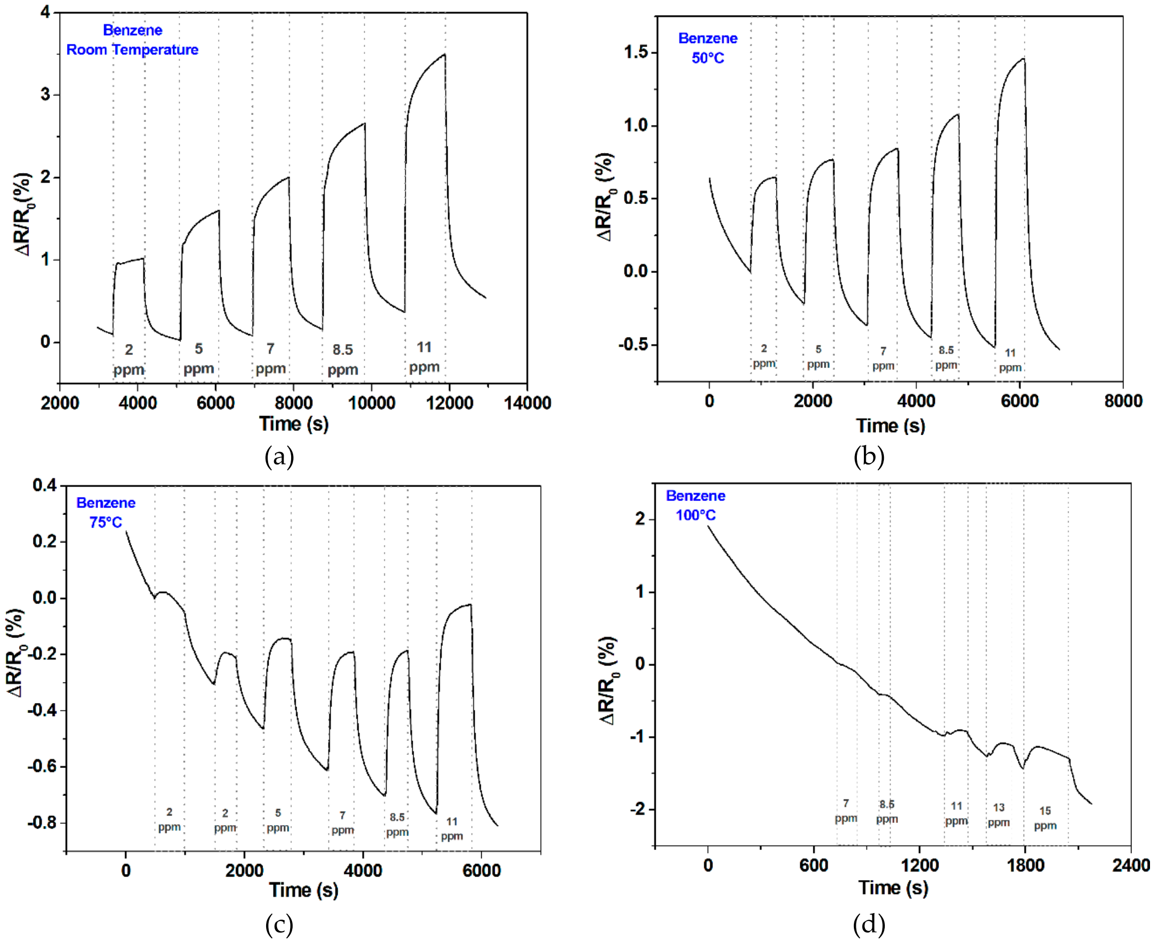

Figure S1. Graphene sensor responses for different concentrations of injected benzene vapors at room temperature, 50 °C, 75 °C and 100 °C.

The table indicates the sensitivity of bare graphene sensors towards the different aromatic VOCs tested as a function of the operating temperature. The best results (in bold) are achieved at room temperature.

Table S1. Graphene sensitivity (10^{-2}x ppm^{-1}) for different vapors at different temperatures.

	Benzene	Toluene	Xylene
**RT***	**19.20**	**91**	**213.6**
**50 °C**	14.93	77.5	196.2
**75 °C**	7.19	58	115.9
**100 °C**	5.75	74.1	60.5

*: Room Temperature

Detection of benzene vapors using metal oxide decorated graphene sensors at different operating temperatures (different from the optimal, which was 250°C).

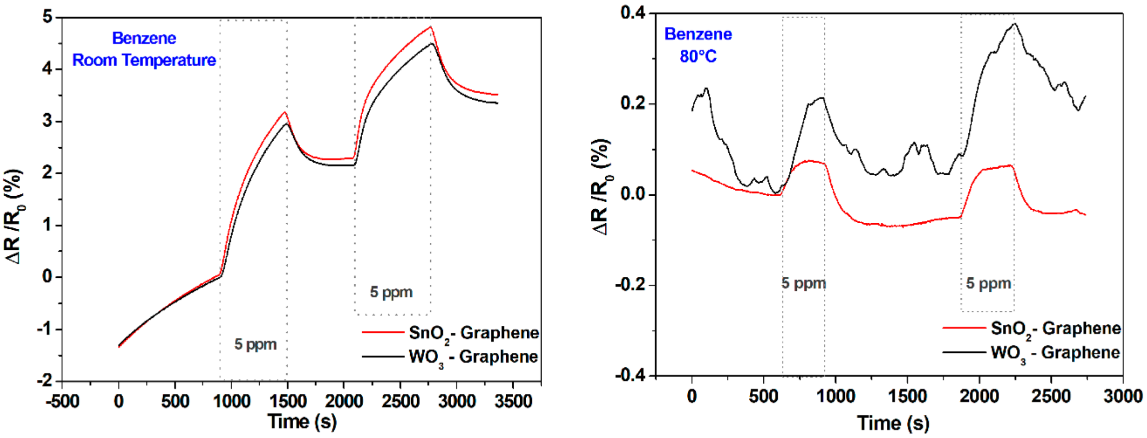

(a) (b)

Figure S2. Decorated Graphene sensors responses for 5 ppm concentration of injected benzene vapor at (a) room temperature and (b) 80°C.

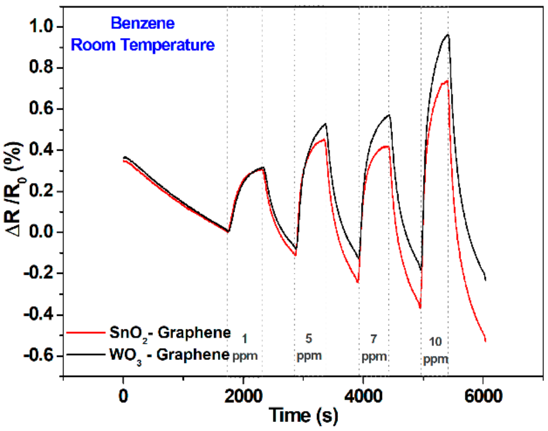

(a)

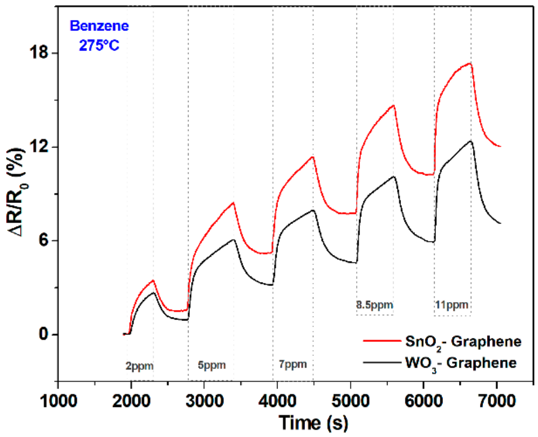

(b)

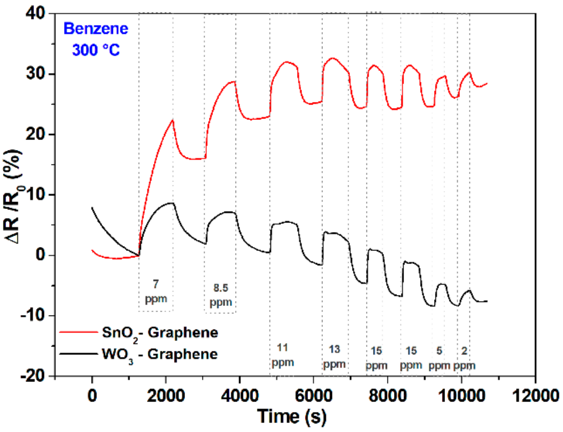

(c)

Figure S3. SnO2-Graphene and WO3-Graphene **s**ensor responses for different concentrations of injected benzene vapors at (a) room temperature, (b) 275 °C and (c) 300 °C.

Detection of toluene vapors using metal oxide decorated graphene sensors at different operating temperatures (different from the optimal, which was 250°C). The results show that a response inversion (from p-type to n-type) occurs when the operating temperature is raised. This happens already at 250°C for tin oxide decorated graphene and at 300°C for tungsten oxide decorated graphene.

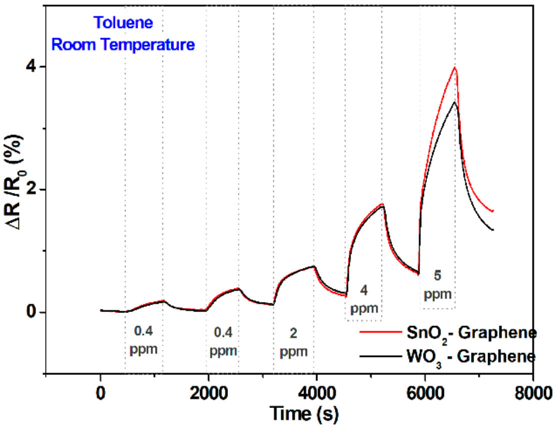

(a)

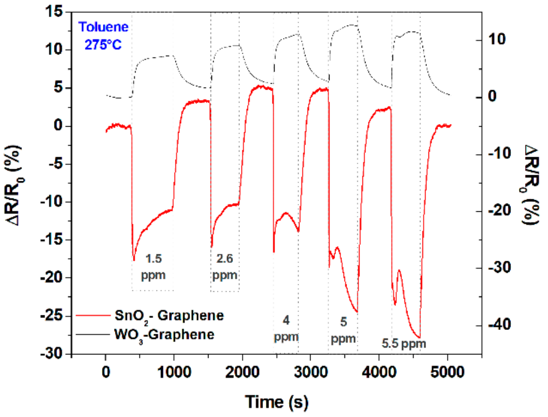

(b)

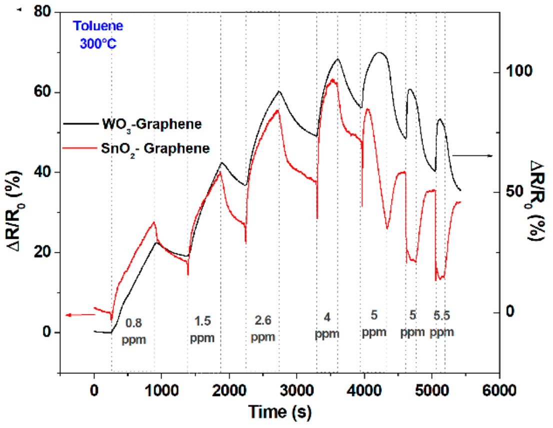

(c)

Figure S4. SnO2-Graphene and WO3-Graphene **S**ensor responses for different concentrations of injected toluene vapors at (a) room temperature, (b) 275 °C and (c) 300 °C.

Detection of o-xylene vapors using metal oxide decorated graphene sensors at different operating temperatures (different from the optimal, which was 250°C). The results show that a response inversion (from p-type to n-type) occurs when the operating temperature is raised for tin oxide decorated graphene.

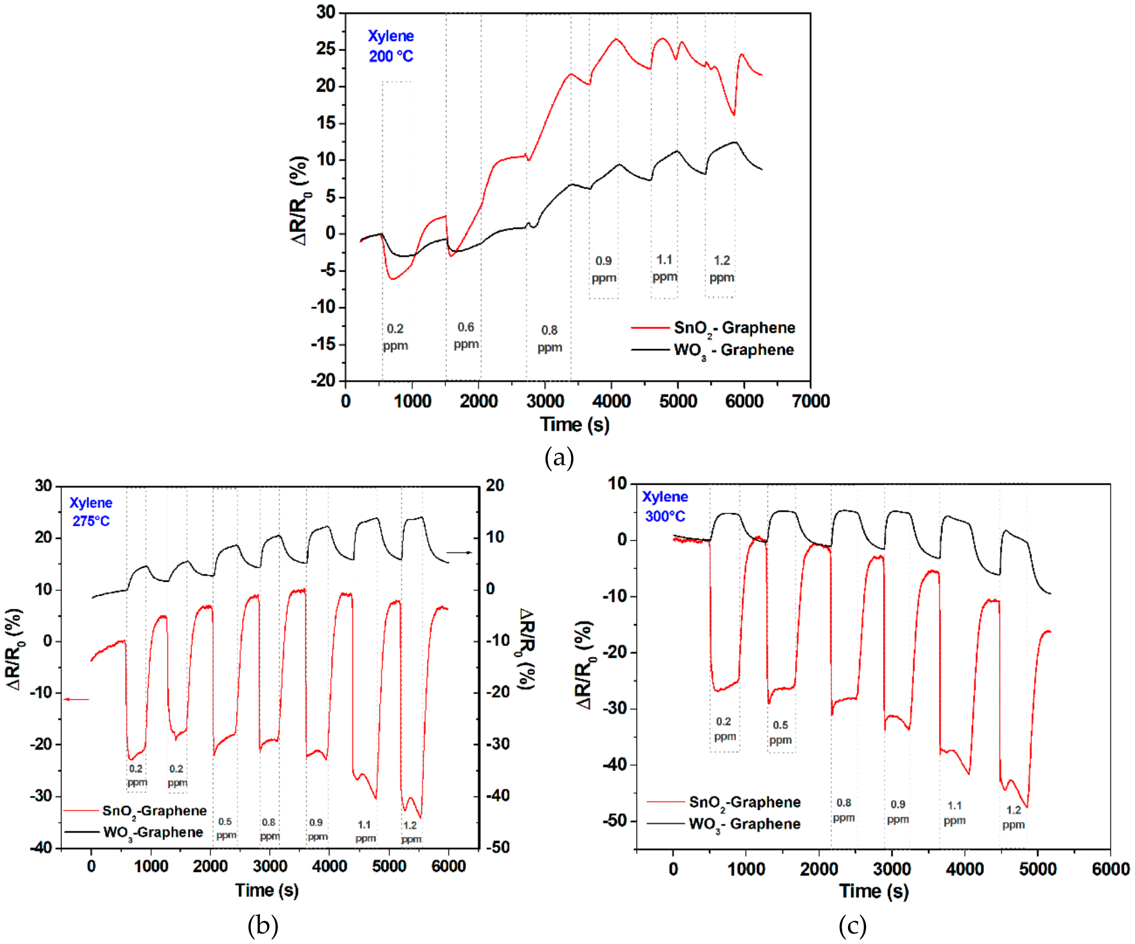

Figure S5. SnO_2-Graphene and WO_3-Graphene **S**ensor responses for different concentrations of injected xylene vapors at (a) 200 °C, (b) 275 °C and (c) 300 °C.

Detection of ethanol vapors using metal oxide decorated graphene sensors at different operating temperatures. The results show that a response inversion (from p-type to n-type) occurs when the operating temperature is raised and sometimes returns to a p-type behavior if the temperature is raised further. This happens at 200°C for tin and tungsten oxide decorated graphene. The p-type behavior is regained for an operating temperature of 300°C.

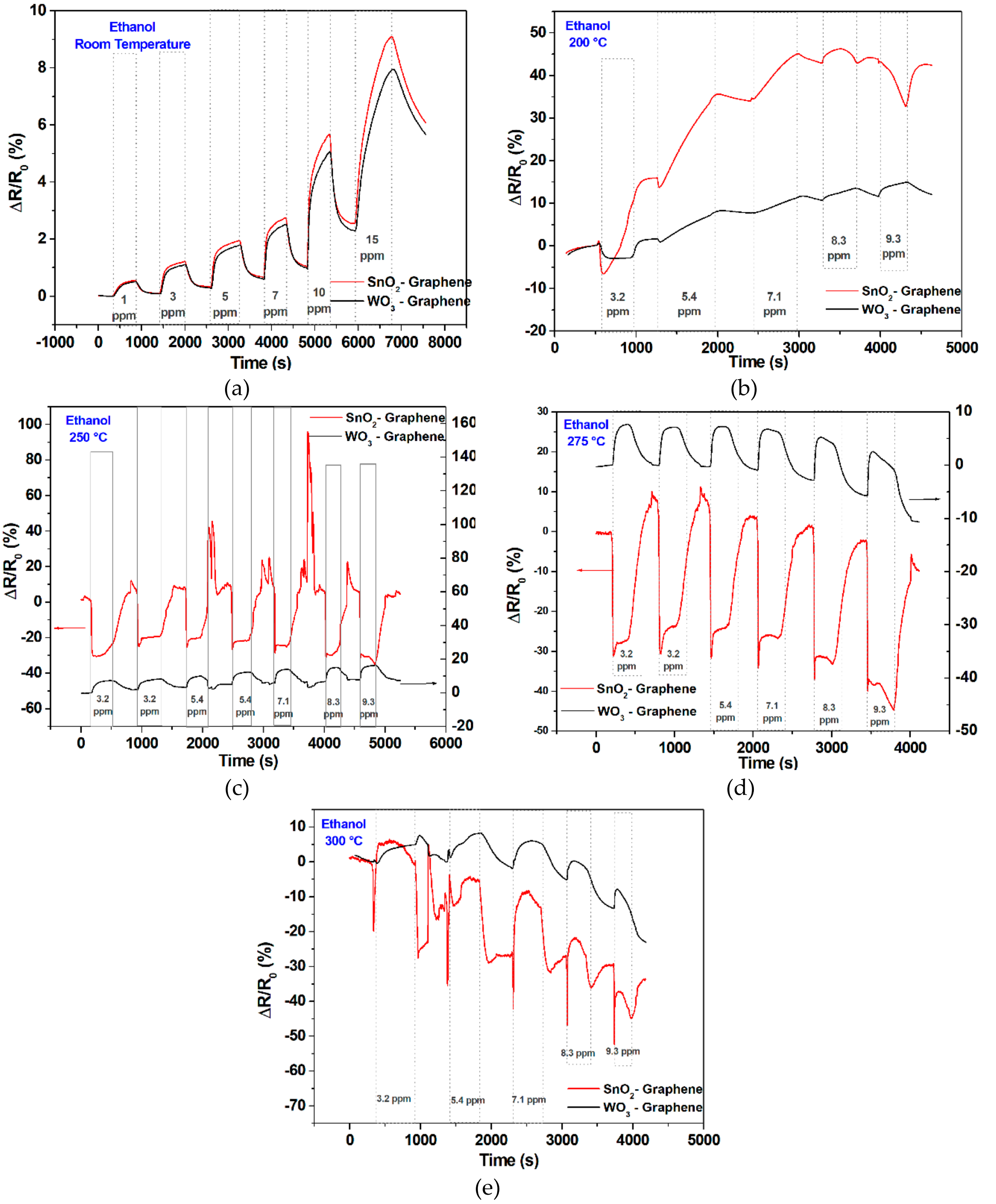

Figure S6. SnO_2-Graphene and WO_3-Graphene sensor responses for different concentrations of injected ethanol vapors. at (a) room temperature, (b) 200 °C, (c) 250 °C, (d) 275 °C and (e) 300 °C.

Table S2. WO_3-Graphene sensitivity (10^{-2}x ppm^{-1}) for different vapors at different temperatures.

	Benzene	Toluene	Xylene	Ethanol
**RT***	8.8	49.0	-	37.9
**250 °C**	**22.7**	**96.9**	**391.2**	23.4
**275 °C**	28.2	60.6	323.7	32.2
**300 °C**	-	302.5	178.1	-

*: Room Temperature

Table S3. SnO_2-Graphene sensitivity (10^{-2}x ppm^{-1}) for different vapors at different temperatures.

	Benzene	Toluene	Xylene	Ethanol
**RT***	9.3	61.0	-	44.4
**250 °C**	**25.5**	**456.4**	**2081.8**	189.6
**275 °C**	33.4	302.6	1662.3	221.3
**300 °C**	-	330.3	1633.3	-

*: Room Temperature

Linear baseline correction

Metal oxide loaded graphene sensors showed a simple, linear baseline drift when they behaved as p-type materials. This was the case of WO_3/graphene sensors for any of the aromatic VOCs tested and also for SnO_2/graphene sensors in the presence of benzene. A typical example of the response data before and after a linear baseline drift correction data has been implemented is presented in the figure below.

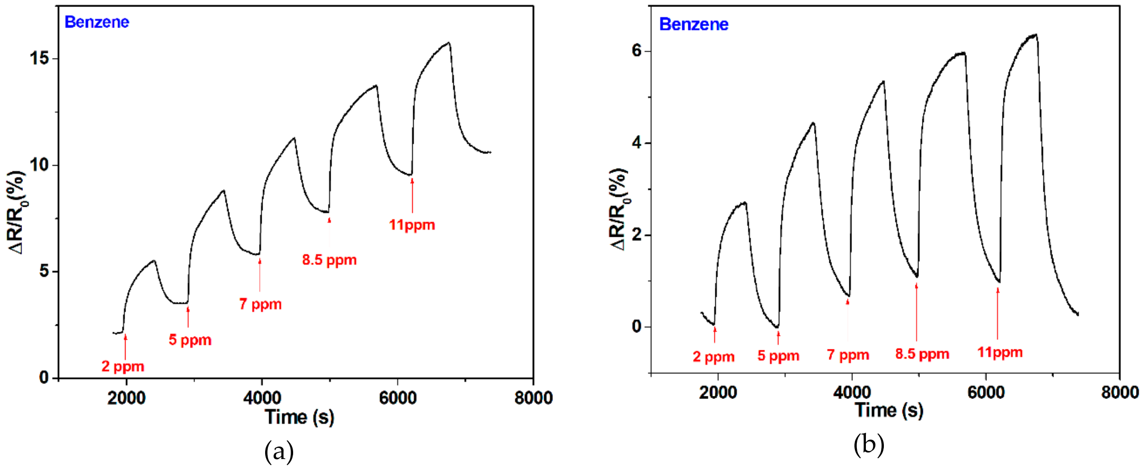

Figure S7: SnO_2-Graphene sensor responses for different concentrations of injected vapors of benzene (a) without and (b) with linear base line correction.

However, SnO_2/graphene sensors in the presence of toluene or xylene show an n-type response behavior. In this particular case, the baseline (see Figure 6 in the main paper) first drifts upwards during the initial concentrations tested to stabilize (case of toluene) or to drift slightly downwards (case of xylene) for the following higher concentrations tested. Since the overall baseline drift is not very high and its behavior is not linear, we preferred not to implement any baseline drift compensation in this case.

Long term stability

The long-term stability of the sensors to benzene vapors was investigated under room temperature and background humidity (60% RH) for a graphene sensor. Response and sensitivity remained stable over the first week of sensor operation, however, a long-term change could be observed. If we consider the calibration curves of the sensors one month after they entered in operation, as presented in Figure S7, a sensitivity drop of about 20 % going from 19.25 10^{-2}x ppm^{-1} to 14.93 10^{-2}x ppm^{-1} was observed. This is possibly due to the adsorption of water molecules on its surface.

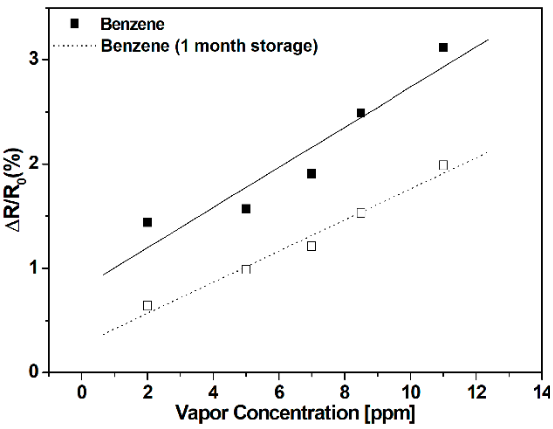

Figure S8. Long-term stability of a graphene sensor to benzene vapors.
